# Invasion and transmission of *Salmonella* Kentucky in an adult dairy herd using approximate Bayesian computation

**DOI:** 10.1186/1746-6148-9-245

**Published:** 2013-12-05

**Authors:** Zhao Lu, Rebecca M Mitchell, Rebecca L Smith, Jeffrey S Karns, Jo Ann S van Kessel, David R Wolfgang, Ynte H Schukken, Yrjo T Grohn

**Affiliations:** 1 Section of Epidemiology, Department of Population Medicine and Diagnostic Sciences, College of Veterinary Medicine, Cornell University, Ithaca, NY 14853, USA; 2 Environmental Microbial and Food Safety Laboratory, Agriculture Research Service, USDA, Beltsville, MD 20705, USA; 3 Department of Veterinary and Biomedical Sciences, Pennsylvania State University, University Park, PA 16802, USA

**Keywords:** Epidemiological modeling, Approximate Bayesian computation, Transmission dynamics, *Salmonella*, Dairy cattle

## Abstract

**Background:**

An outbreak of *Salmonella* Kentucky followed by a high level of sustained endemic prevalence was recently observed in a US adult dairy herd enrolled in a longitudinal study involving intensive fecal sampling. To understand the invasion ability and transmission dynamics of *Salmonella* Kentucky in dairy cattle, accurate estimation of the key epidemiological parameters from longitudinal field data is necessary. The approximate Bayesian computation technique was applied for estimating the transmission rate (*β*), the recovery rate (γ) and shape (*n*) parameters of the gamma distribution for the infectious (shedding) period, and the basic reproduction ratio (*R*_0_), given a susceptible-infectious-recovered-susceptible (SIRS) compartment model with a gamma distribution for the infectious period.

**Results:**

The results report that the mean transmission rate (*β*) is 0.417 month^-1^ (median: 0.417, 95% credible interval [0.406, 0.429]), the average infectious period (*γ*^-1^) is 7.95 months (median: 7.95, 95% credible interval [7.70, 8.22]), the mean shape parameter (*n*) of the gamma distribution for the infectious period is 242 (median: 182, 95% credible interval [16, 482]), and the mean basic reproduction ratio (*R*_0_) is 2.91 (median: 2.91, 95% credible interval [2.83, 3.00]).

**Conclusions:**

This study shows that *Salmonella* Kentucky in this herd was of mild infectiousness and had a long infectious period, which together provide an explanation for the observed prevalence pattern after invasion. The transmission rate and the recovery rate parameters are inferred with better accuracy than the shape parameter, therefore these two parameters are more sensitive to the model and the observed data. The estimated shape parameter (n) has large variability with a minimal value greater than one, indicating that the infectious period of *Salmonella* Kentucky in dairy cattle does not follow the conventionally assumed exponential distribution.

## Background

*Salmonella* is one of the major causes of food-borne gastroenteritis worldwide and poses a considerable threat to public health. In the United States (US) alone there are approximately 1.4 million illnesses, 16,000 hospitalizations, and 400 deaths annually [1,2]. Humans generally acquire salmonellosis through consumption of contaminated food or contact with infected animals or a contaminated environment [3,4]. Emergence of multidrug-resistant *Salmonella* in human infections is particularly serious due to increased morbidity and mortality [5].

More than 2,500 serotypes of *Salmonella* have been identified and significant variability has been found in virulence, infectious dose, and host. Most human *Salmonella* infections are caused by relatively few *Salmonella* subtypes (*S. enterica* Enteritidis, Typhimurium, and NewPort) [6], but all *Salmonella* serotypes are considered potentially pathogenic [7]. Farm animals are recognized as important reservoirs for *Salmonella* and other food-borne pathogens [8]. Many *Salmonella* serotypes have been found in samples from dairy animals and their environment, some of which have also been isolated in human cases [9]. Reduction of *Salmonella* prevalence in farm animals and bacterial loads in the contaminated environment is important to decrease the risk of zoonotic *Salmonella* infection [8].

Aiming to ensure a safe food supply through identifying pathogen transmission pathways and subsequent best management practices in dairy farms, the Regional Dairy Quality Management Alliance (RDQMA) and the Agriculture Research Service (ARS) of the USDA established a longitudinal observational study on three commercial dairy farms in the Northeastern US [10], one of which was located in Pennsylvania. Fecal samples were intensively collected from all adult animals in this herd beginning in the spring of 2004. These samples were tested for *Salmonella* and a number of other food-borne pathogens [10,11]. After an initial outbreak of *Salmonella* Cerro, a subsequent outbreak of *Salmonella* Kentucky with a high level of prevalence and long-term endemic infection in this farm was found [12]. Drug-resistant *Salmonella* Kentucky (ST198) from human cases has recently been identified from a study of National *Salmonella* Surveillance Systems from France, England and Wales, Denmark, and the United States [13]. The identification of *Salmonella* Kentucky is also a common occurrence in poultry [14].

Understanding the transmission dynamics that underlie observed shedding patterns from longitudinal field data is essential for the effective design of *Salmonella* prevention and intervention. As the transmission dynamics of *Salmonella* spp. are determined by complex interactions among host, pathogen, and environment, mathematical modeling approaches have been applied to provide insights in the understanding of transmission [15-19]. However, a common problem in mathematical models in epidemiology is how to accurately and reliably estimate the non-observable model parameters such as the transmission rate, given the available longitudinal field data. To solve this problem, a number of Bayesian inference approaches for infectious disease transmission models have been developed [20-25]. Posterior distributions of parameters can generally be computed using an explicit likelihood function given parameter prior distributions with the help of Markov Chain Monte Carlo (MCMC) methods [20-22], or using a likelihood-free approach, the approximate Bayesian computation (ABC) technique [23] with a newly proposed efficient sequential Monte Carlo algorithm [24,25].

The objective of this study was to infer from longitudinal field data the key epidemiological parameters that are important to understand the invasion ability and transmission dynamics in an outbreak and subsequent endemicity of *Salmonella* Kentucky on a dairy farm. Specifically, we estimated the transmission rate (*β*), the rate (*γ*) and shape (*n*) parameters of the gamma distribution for the infectious (shedding) period, and the basic reproduction ratio (*R*_0_) using the approximate Bayesian computation technique.

## Methods

### Longitudinal field data

The dairy herd (so-called “Farm B”) in Pennsylvania consisted of approximately 100-110 adult cows housed in a free stall barn [10-12]. Calves from this herd were transferred to an off-site rearing center at 6 months of age and were returned to the herd as replacement animals within 1 to 2 months prior to their first calving. Intensive fecal samples were collected for all adult cows in the herd with a sampling interval of 6 to 8 weeks during the study period. Methods for isolation and serotyping of *Salmonella* were previously described [10-12].

The longitudinal data used in this study are composed of observed within-herd prevalence (proportion) of animals shedding *Salmonella* Kentucky for a total of 14 time points from January 2006 to December 2007. As the sensitivity of the culture test for *Salmonella* is generally estimated to be imperfect and relatively low, we used a correction to account for likely false-negative culture results. This rule states that a negative test found between two immediate (neighboring) positive tests in an individual cow was assumed to be a false-negative test and this negative test was corrected to be positive (+- + →+++). The specificity of the culture test for *Salmonella* was assumed to be one, so there were no false-positive tests.

### The SIRS model

Multiple episodes of shedding of *Salmonella* Kentucky were observed in individual cows’ test result profiles. Due to the assumed perfect test specificity and imperfect test sensitivity (see above), at least two or more consecutive negative tests had to appear between these test-positive episodes to define a second or higher shedding (infection) period. On the basis of the shedding pattern of individual cows, we developed a susceptible-infectious-recovered-susceptible (SIRS) transmission model with the following assumptions:

(a) First lactation animals entering the herd as replacement animals were assumed to be susceptible (S) because no positive tests were found in any heifers leaving for the off-site facility or returning to the herd.

(b) Test-positive animals (shedding *Salmonella* Kentucky) were assumed to be in the infectious state (I).

(c) The infectious (shedding) period was assumed to follow a gamma distribution *f*(*t*|*n, γ*) = (*n γ*)^*n*^*t*^*n*-1^ exp (-*nγt*)/Г(*n*)) with two parameters, the rate (*γ*) and shape (*n*), reflecting that time since infection is important [15]. When the shape parameter (*n*) is one, the gamma distribution reduces to an exponential distribution.

(d) Susceptible animals (S) remained in their susceptible state until they became infectious (I) at rate *β I*/ *N*(force of infection), where the transmission rate is denoted by *β*.

(e) The time period covering two or more consecutive negative tests in individual animals between the two neighboring positive tests was relatively long, approximately 3 months or more. Within that time period, animals were assumed to build their immunity, becoming recovered (R). Eventually during that period, the recovered animals (R) lost their immunity and became susceptible (S) again at rate *ϕ*.

(f) Susceptible animals were assumed not to be distinguished based on the presence or absence of prior exposure to *Salmonella* (i.e. no immunological memory).

(g) Direct cow-cow transmission was considered to represent the common fecal-oral transmission route of *Salmonella* Kentucky in the herd.

(h) Herd size was assumed to be a constant (108 cows in this study based on the average herd size across all 14 sampling time points), reflecting a constant size of the milk producing herd.

(i) To maintain a constant herd size, we assumed the replacement rate (*μ*) of the milking herd was the same as the removal rate (*μ*) of animals from the herd.

**Figure 1:**
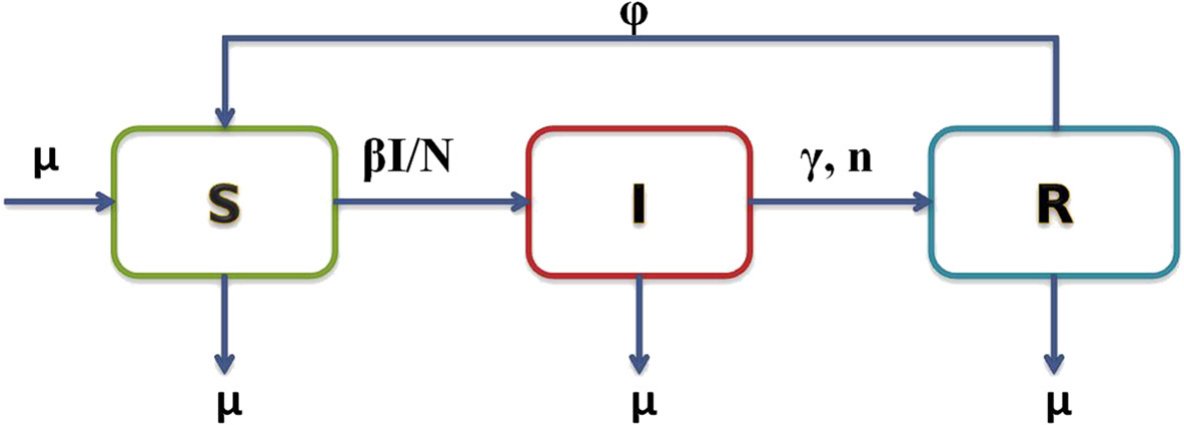
**Flow diagram of the susceptible-infectious-recovered-susceptible (SIRS) model describing the transmission dynamics of *Salmonella *Kentucky in an adult dairy herd.** Susceptible animals (S) were infected at rate *β I* / *N* and became infectious (shedding, I). After remaining infectious a certain amount of time upon infection, the length of which was assumed to follow a gamma distribution described by the rate (*γ*) and shape (*n*) parameters, infectious animals became recovered animals (R), with fully established infection-induced immunity. The recovered animals (R) lost their immunity at rate (*ϕ*) and became susceptible again. To maintain the herd size (N), the replacement rate (*μ*) of animals coming into the herd was assumed to be the same as the general removal rate (*μ*) of animals from the herd.

There are a total of 5 parameters in the SIRS model described in Figure 1. Three of these are unknown and to be estimated: the transmission rate (*β*) and the rate (*γ*) and shape (*n*) parameters describing the gamma distribution for the infectious period. The replacement rate (*μ*, or the removal rate) of animals was assumed to be constant and was calculated from the animal movement data to be 0.03 (month)^-1^, representing the average duration of survival of cows in the milking herd at approximately 2.8 years. The rate of immunity loss (*ϕ* = 0.33 (month)^-1^) was also assumed constant and calculated to be around 3 months because it needed to be equal to or less than the time period for at least two consecutive negative tests (covering the recovered (R) state and its next susceptible (S) state).

The system of ordinary differential equations describing the transmission dynamic model (SIRS) with a gamma distribution for the infectious period in Figure 1 is given in Additional file 1: Appendix A.

### Method of estimating parameters

We applied the approximate Bayesian computation (ABC) technique [23] to infer the unknown epidemiological parameters (*β*, *γ*, *n* and *R*_0_) given the longitudinal data and the SIRS model. Uniform (flat) prior distributions were assumed for the 3 unknown parameters, *β* ∈ [0.01,2], *γ* ∈ [0.01,1], and n ∈ [1, 500].

Unknown parameters (*β*, *γ*, *n*) were sampled from their prior distributions and these sampled values were used to numerically solve the system of ordinary differential equations (the SIRS model in Additional file 1: Appendix A). The sum of squared errors between the fitted and observed prevalence was calculated. If the sum of squared errors was less than a desired tolerance value, then the sampled parameter values were accepted. However this rejection algorithm was not effective and was not able to put into in practice due to high computational demands. An efficient algorithm recently developed for ABC using the sequential Monte Carlo method was implemented for parameter estimate and model selection for nonlinear dynamic systems [24,25]. In this study we used this efficient algorithm for parameter estimation.

To examine the invasion ability, we estimated the basic reproduction ratio (*R*_0_), which represents the number of secondary cases caused by the introduction of a primary index case into a fully susceptible population during its whole infectious period. The basic reproduction ratio (*R*_0_) of the SIRS model in this study is a function of the other three parameters (*β*, *γ*, *n*)and was obtained using the next-generation matrix [26]:

(1) $$R_{0}=\frac{\beta }{\mu }\left(1-{\left(\frac{\mathrm{n\gamma}}{\mathrm{n\gamma}+\mu }\right)}^{n}\right)$$

### Posterior predictive check and cross-validation

Using the estimates from the posterior distributions of parameters (*β*, *γ*, *n*), stochastic simulations based on the direct Gillespie algorithm for the SIRS model were performed [27]. The expected prevalence and its 2.5% and 97.5% quantiles were compared to the observed prevalence. As we had only one dataset of the observed longitudinal prevalence (consisting of 14 data points), cross-validation was performed to check the behavior of the SIRS model. A total of 14 datasets for cross-validation were formed by removing each data point once from the full observed prevalence data set.

### Impact of the rate of loss immunity

As the rate at which immunity wanes (*ϕ*) was uncertain and partly based on the sampling interval, we repeated the ABC analyses with a slower rate, 0.25 (month)^-1^, to investigate the effects of varying the rate of immunity loss (*ϕ*) on the estimation of the transmission rate (*β*), the rate (*γ*) and shape (*n*) parameters for the gamma distribution for the infectious period, and the basic reproduction ratio (*R*_0_).

## Results

### Posterior distributions of parameters (β, γ, n)

**Figure 2:**
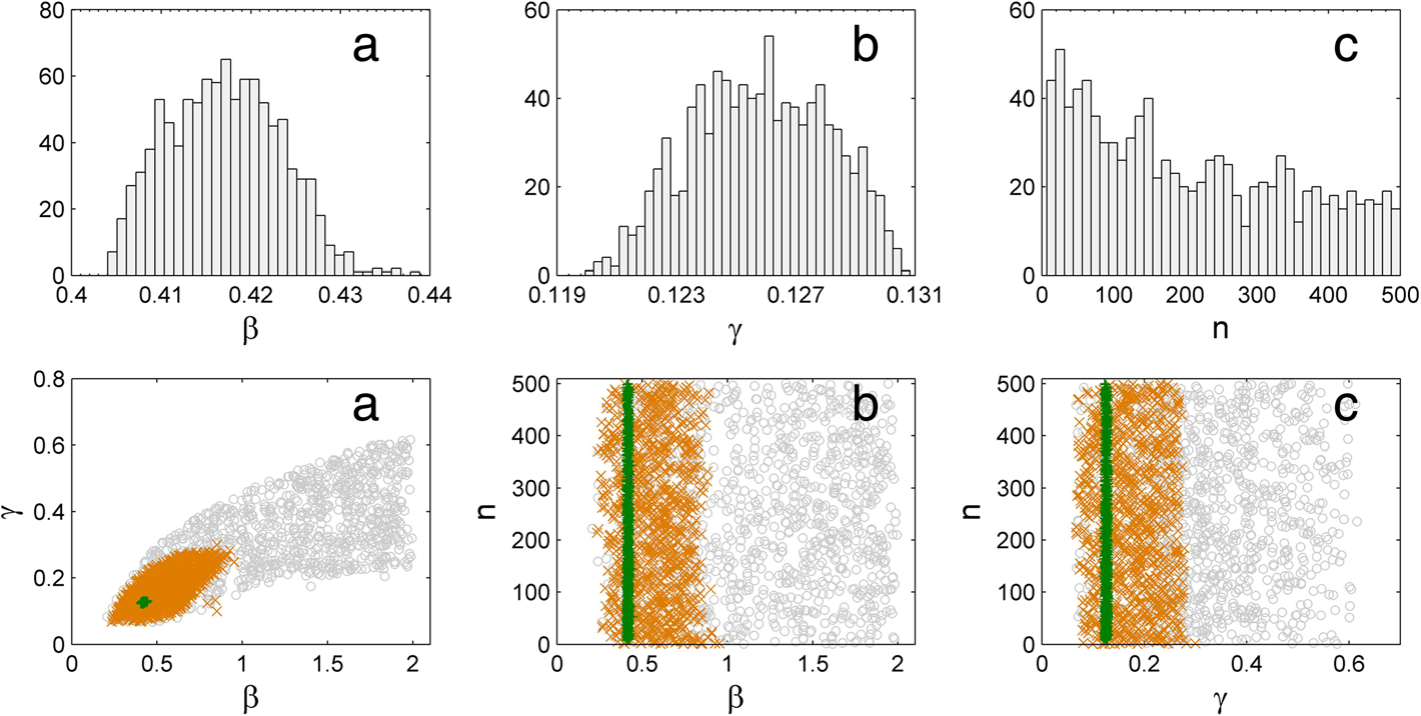
**Posterior distributions (top row: a, b, c) and scatter plots (bottom row: a, b, c) of the transmission rate (*β*), and the rate (*γ*) and shape (*n*) parameters for the gamma distribution for the infectious period.** Each scatter plot shows three time snapshots from the start of simulation to the final stable state, illustrating the process of how the sequential Monte Carlo algorithm in approximate Bayesian computation results in the convergent posterior distribution.

Posterior distributions of the transmission rate (*β*), and the rate (*γ*) and shape (*n*) parameters of the gamma distribution describing the infectious period are shown in the top row of Figure 2. All distributions were unimodal. For the transmission rate (*β*) and the rate of recovery (*γ*) the ranges of these two posterior distributions were relatively narrow. The transmission rate (*β*) showed a mean of 0.417 month^-1^ with a 95% credible interval of 0.406 to 0.429. The average recovery rate (γ) was 0.126 month^-1^ with a 95% credible interval of 0.1216 to 0.1298 month^-1^. The shape parameter (*n*) had large variability with a median of 182 (mean: 242) with a 95% credible interval of 16 to 482.

The bottom row of Figure 2 shows scatter plots of paired parameters. Each scatter plot illustrates three time snapshots from the start of simulation to the middle and finally to the stable state – the process of how the sequential Monte Carlo algorithm in ABC leading to the convergence posterior distribution.

### The infectious period and the basic reproduction ratio (R_0_)

**Figure 3:**
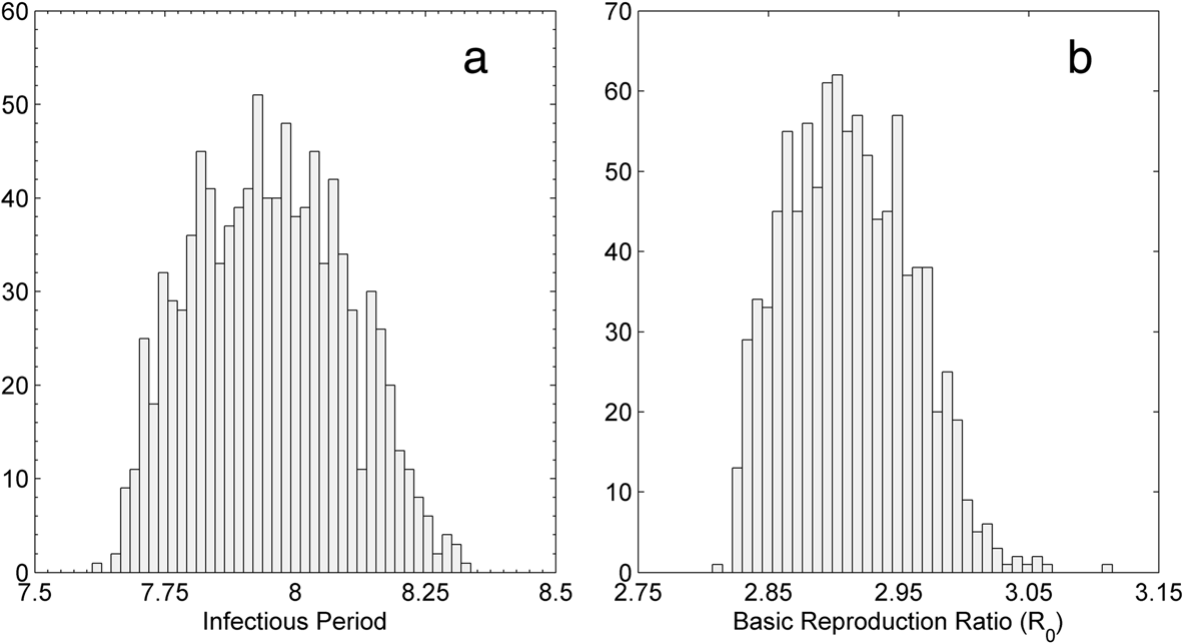
**Distributions of the infectious period and the basic reproduction ratio (*R***_**0**_**) estimated from the posterior distributions of the transmission rate (*β*), the rate recovery (*γ*) and shape (*n*) parameters.**

The distributions of the infectious period and the basic reproduction ratio (*R*_0_) using the estimates from the posterior distributions of parameters (*β*, *γ*, *n*) are shown in Figure 3a and 3b, respectively. The average infectious period had a mean of 7.95 months (1/*γ =* 1/ 0.1258) with a 95% credible interval of 7.70 (1/*γ =* 1/ 0.1298) to 8.22 (1/*γ =* 1/ 0.1216) months. The *R*_0_ had a mean of 2.91 with a 95% credible interval of 2.83 to 3.00.

### Posterior predictive check

**Figure 4:**
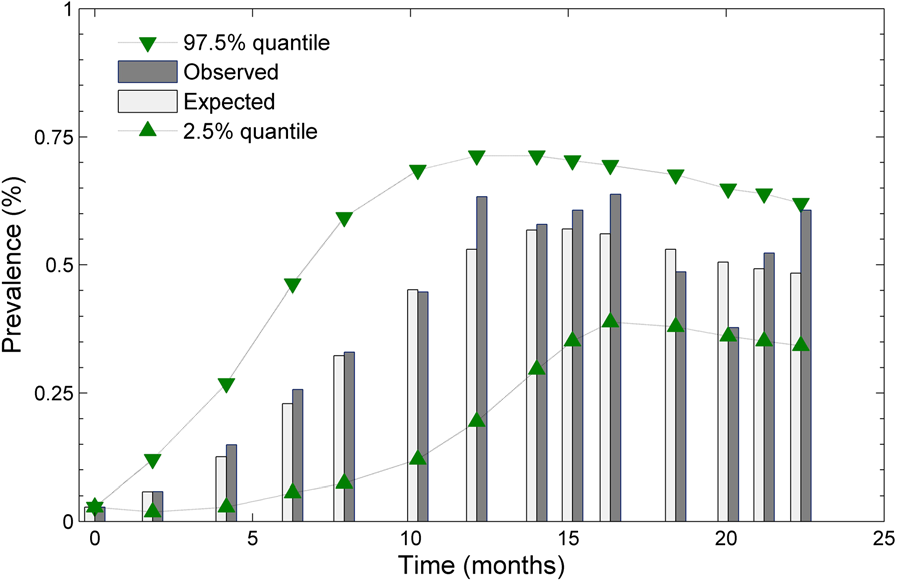
**Comparison of the observed prevalence with the predicted mean 2.5% quantile and 97.5% quantile prevalence for *Salmonella *** **Kentucky over approximately 2 years.**

Comparison between the observed prevalence and the expected (fitted) prevalence with the predicted 2.5% and 97.5% quantile is demonstrated in Figure 4. The expected prevalence exhibited agreed well with the observed prevalence before *Salmonella* Kentucky reached endemic prevalence. All of the observed prevalence data points were within the range from the predicted 2.5% to 97.5% quantile; two observed prevalence data points, the 12^th^ and 14^th^, were quite close to the low 2.5% and high 97.5% quantiles, respectively.

### Cross-validation

**Figure 5:**
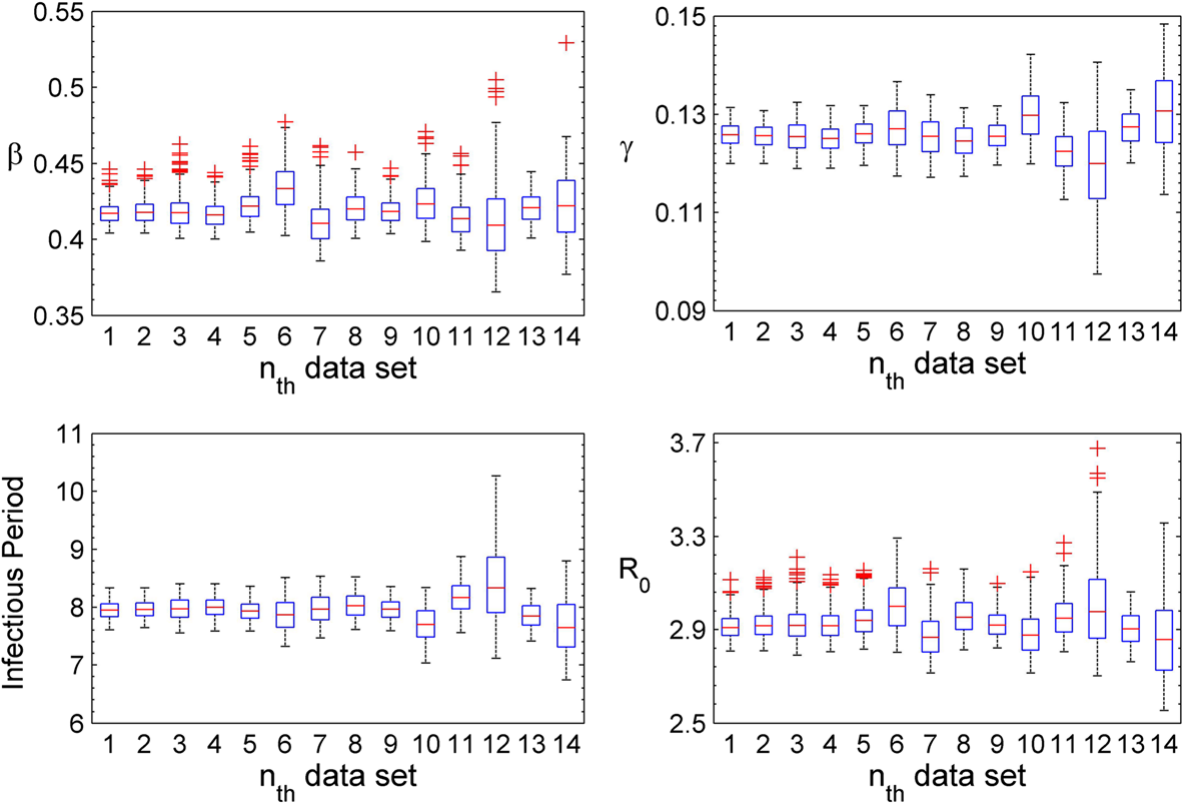
**Estimates of the transmission rate (*β*), the recovery rate (*γ*), the infectious period, and the basic reproduction ratio (*R***_**0**_**) against all 14 datasets (with the i**^**th **^**observed data point removed) used for the cross-validation of the SIRS model.**

Boxplots of the transmission rate (*β*), the recovery rate (*γ*), the infectious period, and the basic reproduction ratio (*R*_0_) are shown for 14 sub-datasets in Figure 5. The transmission rates (*β*) mostly ranged from 0.39 to 0.46 month^-1^, and the recovery rate parameters (*γ*) from 0.112 to 0.138 month^-1^. The infectious period was mostly between 7 and 9 months, and the *R*_0_ value was between 2.70 and 3.14 for almost all datasets.

### Impact of varying the rate of immunity

We studied the impacts of varying the rate of immunity (*ϕ*) on the transmission rate (*β*), the rate (*γ*) and shape (*n*) parameters of the gamma distribution for the infectious period, and the basic reproduction ratio (*R*_0_). When the rate of immunity (*ϕ*) was changed from 0.33 (month)^-1^ to 0.25 (month)^-1^ (i.e., the average duration in the recovery state (R) thereby increased from 3 months to 4 months), the estimated transmission rate (*β*) parameter (mean: 0.417 → 0.428 month^-1^; 95% credible interval [0.1216, 0.1298] → [0.1137, 0.1249] month^-1^), and the rate of recovery (*γ*) parameter (mean: 0.1258 → 0.1195 month^-1^; 95% credible interval [0.1216, 0.1298] → [0.1137, 0.1249] month^-1^), and the basic reproduction ratio (*R*_0_) (mean: 2.9 → 3.09; 95% credible interval [2.83, 3.00] → [2.91, 3.31]) changed only slightly, but the shape parameter (*n*) had a relatively large change and was well inferred (median: 182 → 11; 95% credible interval [16, 482] → [4, 32]). Posterior distributions of these parameters are given in Figure B1 in Additional file 1: Appendix B.

## Discussion

The mean estimate of the basic reproduction ratio (*R*_0_) for *Salmonella* Kentucky in adult dairy cows from our data is 2.91, which indicates a relatively mild infectiousness (one primary shedding cow can, on average, infect 3 susceptible cows). The relatively modest *R*_0_ value also indicates that preventative efforts to reduce the reproduction rate to values below 1 may be reasonable and even so-called ‘leaky’ vaccines may prove to be sufficiently efficacious to provide herd immunity [17].

The mean duration of the infectious (or shedding) period is 8 months. This estimated duration is long compared to other *Salmonella* strains causing clinical signs in dairy cattle, such as *Salmonella* Typhimurium and *Salmonella* Dublin [18]. The mild infectiousness and long infectious period together explained the observed dynamic pattern of *Salmonella* Kentucky in Figure 4; the prevalence of animals shedding *Salmonella* Kentucky gradually increased during the epidemic phase and then a relatively stable long-term endemic infection was established in the herd.

The posterior distribution of the shape parameter (*n* = 182, 95% credible interval [16, 482]) of the gamma distribution for the infectious period did not include *n* = 1; therefore, the conventional assumption that the infectious period is described by an exponential distribution was found to be inappropriate and a more realistic gamma distribution for the infectious period was favored. This indicates that time since infection is important in the transmission dynamics of *Salmonella* Kentucky in adult dairy cattle. Consequently, strategies for prevention and intervention could be affected by the infection time of animals in the herd [28]. Although large variability of the shape parameter is observed with an assumption of a 3-month period of immunity loss, it can be significantly reduced if a 4-month period of immunity loss is assumed (Additional file 1: Appendix B).

We expect that for many more persistent infections, the assumption of an exponential rate of disappearance from the infectious state will be incorrect. Implementation of non-exponential distributions in ODE-based parameter estimation is not straightforward. The ability to use distributions other than exponential, with the gamma distribution being an attractive alternative, may be one of the key benefits of using ABC for parameter estimation, as no explicit likelihood function needed to be defined. This was especially helpful in this study, where we used a gamma distribution for the infectious period. However, the lack of an explicit likelihood function requires highly demanding computational efforts. Therefore, the implementation of an efficient algorithm in ABC as shown here became imperative.

Posterior distributions of the transmission rate and the rate of recovery (*β*, *γ*) obtained from ABC (Figure 2) were relatively narrow. This was also described in a previous study [24], partly due to the use of a deterministic model (a system of differential equations) in the simulation of ABC. If a stochastic SIRS model (in the formulation of either stochastic differential equations or continuous-time Markov Chain) was used in ABC, the credible intervals would be wider due to the addition of stochasticity into the SIRS model. The transmission rate (*β*) and the recovery rate (*γ*) parameters had significantly better accuracy than the shape parameter (n) as shown in the top row of Figure 2, which indicated that these two parameters were more sensitive to the model and data than the shape parameter (n) [24].

We did not apply ABC to the stochastic SIRS model because the estimated values of the transmission rate (*β*), the rate of recovery (*γ*) and the shape parameter (*n*) from the deterministic SIRS model were sufficient to capture the observed prevalence pattern (Figure 4) using a stochastic SIRS model implementing the direct Gillespie algorithm. We also did not perform elaborate model selection in this study because the SIRS model appears to be capable of explaining the observed transmission dynamics of *Salmonella* Kentucky. As mentioned before, the observed intermittent shedding may be explained by the relatively poor sensitivity of culture methods and we therefore corrected for the occasional assumed false-negative result. Further research to distinguish between assuming true intermittent shedding [29] and a non-perfect test sensitivity in continuous shedding may be necessary.

The posterior predictive check shown in Figure 4 indicated that the estimated transmission rate (*β*) and the rate (*γ*) and shape (*n*) parameters in Figure 2 were reasonable, as stochastic simulations for the SIRS model with these parameter estimates from their posterior distributions were able to capture the observed dynamic (prevalence) pattern. The cross validation (Figure 5) suggested that the estimate of the basic reproduction ratio (*R*_0_) was fairly consistent. Although the shape parameter had large variability (the top row of Figure 2c), it did not have a substantial effect on the basic reproduction ratio. In other words, neither the basic reproduction ratio (*R*_0_) nor the dynamic pattern were sensitive to changes in the shape parameter (n).

When varying the period of immunity from 3 months to 4 months, no significant changes were found in the transmission rate and the rate of the gamma distribution for the infectious period. However, serological data that are able to distinguish the recovered state (R) and the susceptible (S) state would help remove the uncertainty in the rate of immunity loss and increase the accuracy of parameter estimation, especially for the estimation of the shape parameter (n).

## Conclusions

We developed a susceptible-infectious-recovered-susceptible (SIRS) model to describe the transmission of *Salmonella* Kentucky in an adult dairy herd. The important epidemiological parameters of the SIRS model were estimated from a longitudinal data set using the approximate Bayesian computation method. This study shows that *Salmonella* Kentucky has a mild invasion ability (*R*_0 =_ 2.91, 95% credible interval [2.83, 3.00]) and has a long average infectious period (7.95 months, 95% credible interval [7.70, 8.22]) in dairy cattle. These findings together provide an explanation for the observed prevalence pattern after invasion. The transmission rate and the recovery rate parameters are inferred with better accuracy than the shape parameter, therefore these two parameters are more sensitive to the model and the observed data. The estimated shape parameter (n) has large variability with a minimal value greater than one, indicating that the infectious period of *Salmonella* Kentucky in dairy cattle does not follow the conventionally assumed exponential distribution.

## Competing interests

The authors declare that they have no any competing interests.

## Authors' contributions

ZL, RMM, YHS and YTG conceived the study. YHS, YTG, ZL, RMM, and RLS contributed to the model development. ZL implemented the model and performed parameter estimation. JSK, JASVK, and DRW collected the field data and analyzed the field prevalence. All authors contributed to the writing of the manuscript, and read and approved the final version of the manuscript.

## Supplementary Material

Additional file 1 **Appendix A.** Appendix B. Click here for file
