# Supplementary material for: Invasion and transmission of Salmonella Kentucky in an adult dairy herd using approximate Bayesian computation
Source: BMC Vet Res. 2013 Dec 5;9:245. doi: 10.1186/1746-6148-9-245 (PMC4235045; doi:10.1186/1746-6148-9-245)
Supplement: Additional file 1 — Appendix A. Appendix B. [file 1746-6148-9-245-S1.doc]

**Appendix A: The system of ordinary differential equations with a gamma distribution for the infectious period**

The deterministic model of the SIRS model (Fig. 1) for *Salmonella* Kentucky in a dairy herd was described by the following ordinary differential equations:

(A1)

The gamma distribution for infectious period was mathematically decomposed into a sequence of n-stage infectious compartments, from $I_{1}$to$I_{n}$ . In this study, the first sampling time on January 31, 2006 was reset as the initial time ($t=0$). A constant herd size,$N=108$, was assumed, and the initial number of infectious (shedding) animals ($I_{t=0}=3$) was assumed to be the number of test-positive (shedding) animals from the first observed data point (Jan. 31, 2006, t=0). The system of the ordinary differential equations (A1) for the SIRS model was numerically solved with the initial condition$S_{t=0}=105$, $I_{t=0}=3$, and $R_{t=0}=0$. The algorithm using the efficient sequential Monte Carlo method in ABC described in a previous study was implemented to estimate parameters.

The distance (tolerance) function used in the ABC-SMC implementation was defined as the square root of the sum of squared distance between the observed prevalence and the model predicted prevalence over each time point:

$d=\left(\sum_{i=1}^{14} \left(Y_{obs,i}-Y_{pred,i}\right)^{2}\right)^{1/2}$*.*

To set an appropriate tolerance value for calculating the credible intervals of three model parameters ($\beta$,$\gamma$, and $n$), we did the following steps. First, the best estimates of these three parameters were obtained by setting the tolerance at the lowest tolerance value possible. In our study this tolerance value is 0.2130 and the best estimates for gamma and n are 0.125 month^-1^ and 16, respectively. Second, the infectious period follows a gamma distribution with scale parameter ($n\times\gamma=2$) and shape parameter (n=16), so the standard deviation for the infectious period is 2. Third, we used additional information to calculate the standard error, $2/\sqrt{237}$, where the sample size (237) is the number of infectious period observed in our data set during the study period, Therefore, the 95% confidence interval for the infectious period would vary roughly between 7.74 to 8.26 months, which means that the 95% confidence interval for the recovery rate ($\gamma$) should be approximately between 0.1211 to 0.1292 month^-1^. Fourth, using the information from step 3, we reset the tolerance value as 0.2186 (instead of the lowest tolerance value possible 0.2130) in order to get credible intervals for model parameters. With the same tolerance value, we ran the ABC-SMC for other 13 cross validation datasets as well. All calculations were performed using *MATLAB®* software (MathWorks, 2010a).

**Appendix B: Posterior distributions of model parameters ($\beta$,$\gamma$, and $n$) and distributions of the infectious period and the basic reproduction ratio when the rate of immunity loss () is 0.25 month^-1^.**

We applied the same approach as described in Appendix A to find the appropriate tolerance value for inferring model parameters ($\beta$,$\gamma$, and $n$) in the case of the rate of immunity becoming 0.25 month^-1^. The best estimates of the recovery rate ($\gamma=0.1194$) and the shape parameter ($n=8$) were obtained by setting the tolerance value to 0.2226. The standard error for the distribution of the infectious period is 0.192. Thus, the 95% confidence interval for the infectious period would vary roughly between 7.998 and 8.752 months, which suggests that the 95% confidence interval of the recovery rate ($n$) parameter should be between 0.1143 and 0.1250 month^-1^. The appropriate tolerance value was then determined to be 0.2263. We reset the tolerance value to 0.2263 for ABC-SMC to find the posterior distributions of model parameters and the distributions of infectious period and the basic reproduction ratio, which are shown in the following two figures.
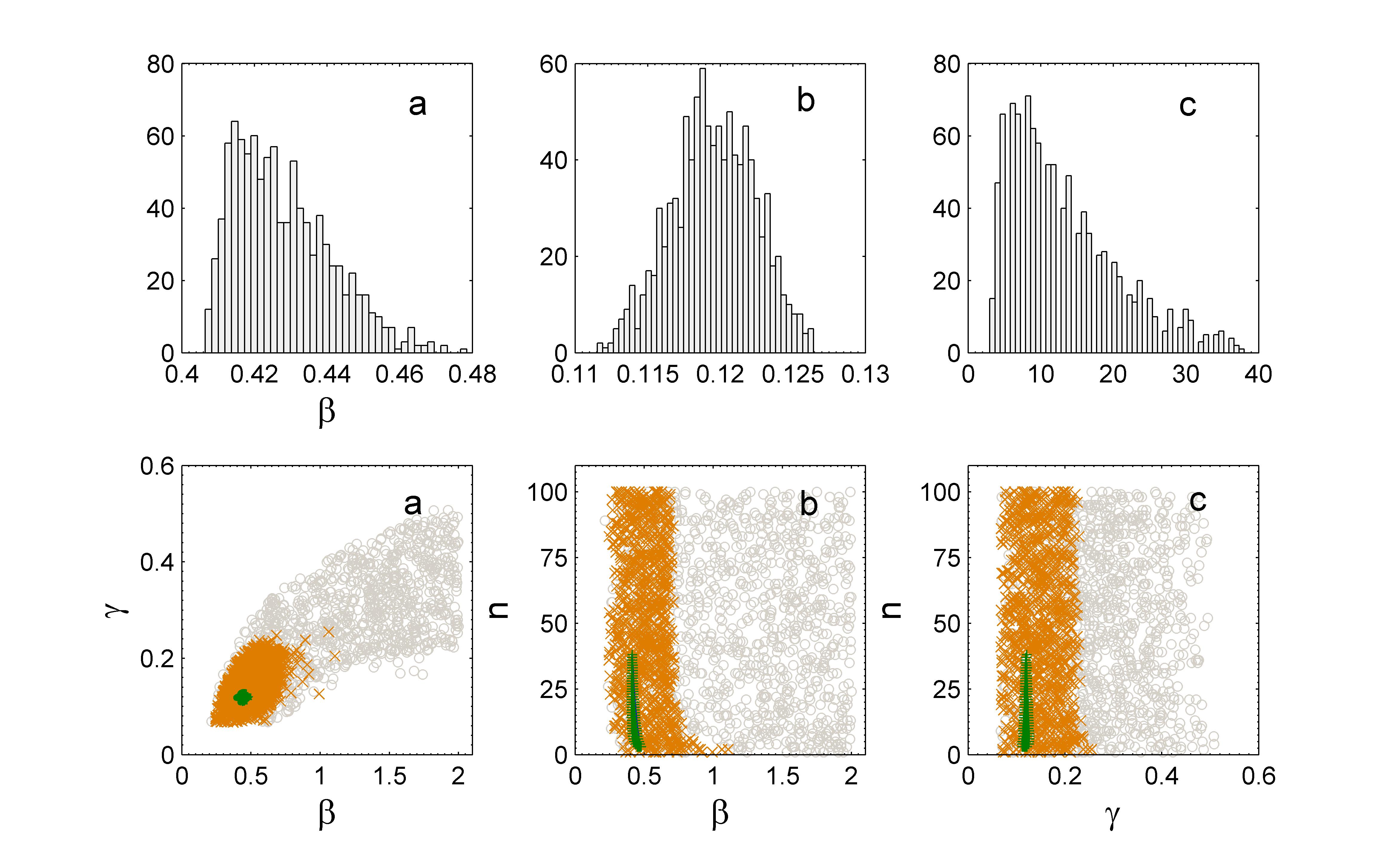


Figure B1: Posterior distributions (top row) of the transmission rate ($\beta$, mean: 0.428, median: 0.425, 95% credible interval [0.409, 0.458] month^-1^), the recovery rate ($\gamma$, mean: 0.1195, median: 0.1195, 95% credible interval [0.1137, 0.1249] month^-1^), and the shape parameter ($n$, mean: 13, median: 11, 95% credible interval [4, 32]). Scatter plots (bottom row) of paired parameters; each scatter plot shows three time snapshots from the start of simulation to the final stable state, illustrating the process of how the sequential Monte Carlo algorithm in approximate Bayesian computation results in the convergent posterior distribution.


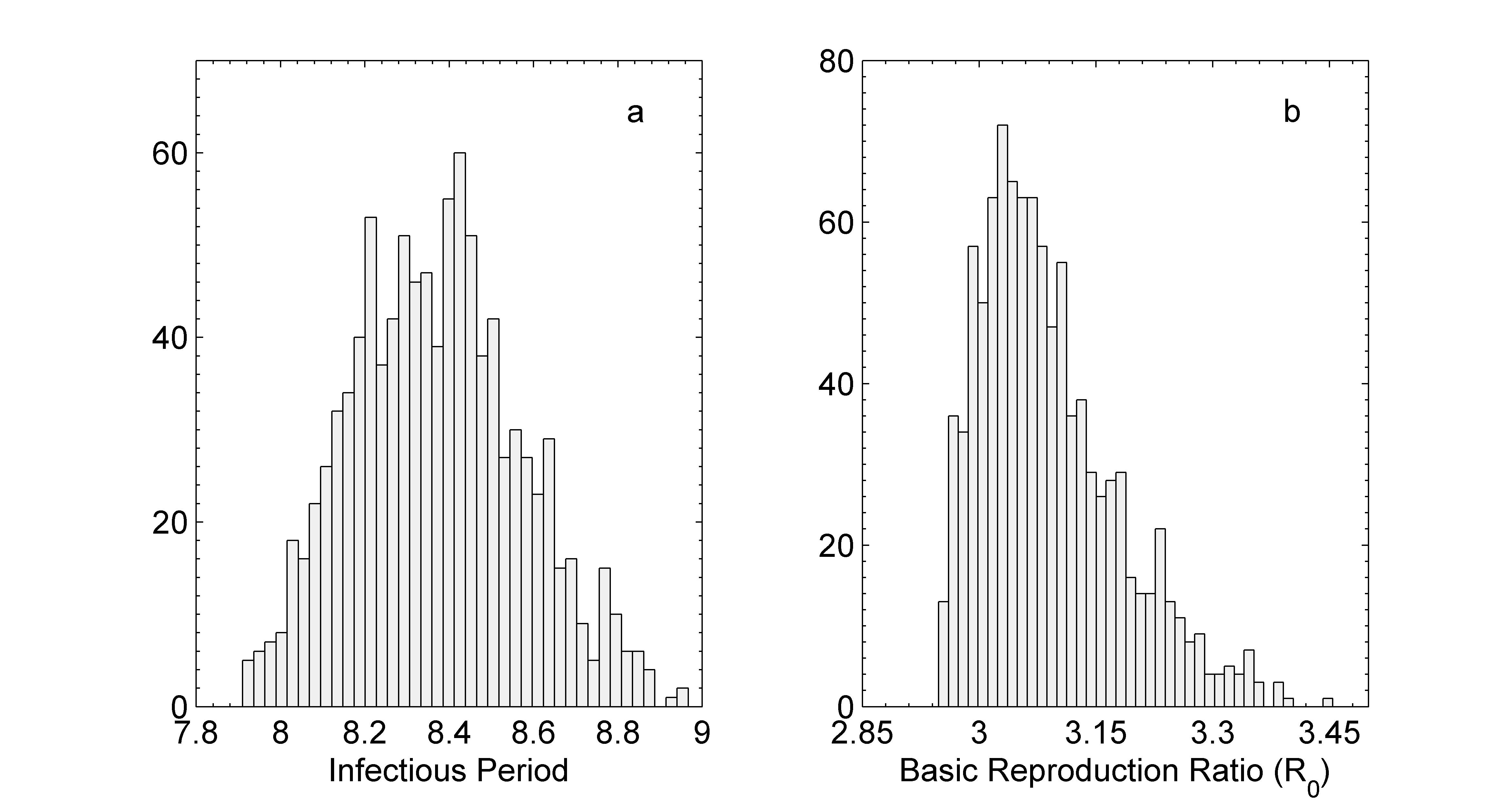


Figure B2: Distributions of the infectious period (mean: 8.368, median: 8.366, 95% credible interval [8.008, 8.794] month) and the basic reproduction ratio ($R_{0}$, mean: 3.08, median: 3.07, 95% credible interval [2.97, 3.31]) estimated from the posterior distributions of the transmission rate ($\beta$), the rate recovery ($\gamma$) and shape ($n$) parameters.
